# Targeting physical activity promotion in general practice: Characteristics of inactive patients and willingness to change

**DOI:** 10.1186/1471-2458-8-172

**Published:** 2008-05-22

**Authors:** Gonzalo Grandes, Alvaro Sánchez, Jesús Torcal, Ricardo Ortega Sánchez-Pinilla, Kepa Lizarraga, Javier Serra, The PEPAF Group

**Affiliations:** 1 Primary Care Research Unit of Bizkaia, Basque Health Service, Bilbao, Spain; 2 Basauri-Ariz Health Centre, Basque Health Service, Basauri, Spain; 3 Santa Bárbara Health Centre, Castilla La Mancha Health Service, Toledo, Spain; 4 Sports Medicine Service of the Bizkaia's Provincial Administration, Bilbao, Spain; 5 Preventive Services and Health Promotion Research Network -redIAPP-, Spain; 6 Unidad de Investigación de Atención Primaria de Bizkaia, Osakidetza, Luis Power 18, 4a planta. 48014 – Bilbao, Spain

## Abstract

**Background:**

Counselling in routine general practice to promote physical activity (PA) is advocated, but inadequate evidence is available to support this intervention, and its sustainable implementation over time is difficult.

**Objectives:**

To describe the characteristics of physically inactive adults visiting GPs and the factors associated with their willingness to change PA.

**Methods:**

A cross-sectional analysis of 4317 Spanish people aged 20–80 years, selected by systematic sampling among those attending 56 public primary health care practices identified as inactive by their GPs in 2003. PA (7-day PAR), PA stage of change, health-related quality of life (SF-36), cardiovascular risk factors, and social and demographic characteristics were measured. Multivariate mixed effects ordinal logistic models were adjusted to identify factors associated with motivational readiness to change.

**Results:**

At least 70% (95% CI: 67.6% to 72.8%) of patients assessed by GPs did not achieve minimal PA recommendations. In addition, 85% (95% CI: 83% to 86.3%) had at least an additional cardiovascular risk factor. Only 30% (95% CI: 25.8% to 33.5%) were prepared for or attempting a change. A younger age; retirement or work at home; higher education and social class levels; obesity; and hypertension were associated with a higher motivational readiness to change (p < 0.05).

**Conclusion:**

The overburden that would result from counselling such a high proportion of inactive primary care patients justifies a targeted strategy for PA promotion in family practice. Selection of a target population based on readiness to change, the combination of risk factors and socio-demographic characteristics of patients is suggested in order to prioritise promotion efforts.

## Background

Physical inactivity is an independent risk factor for cardiovascular disease, diabetes, hypertension, obesity, osteoporosis, colon, breast and other cancers, depression, anxiety and other illnesses [1,2]. However, in developed countries a majority of the population does not reach the minimal recommended levels of physical activity (PA) [1-4]. Primary care practitioners can take advantage of the ongoing care they provide to a large sector of the population, that offers multiple opportunities to briefly advise and assist inactive patients over the long term [5]. Accordingly, PA evaluation and advice is recommended in primary care patients [6]. However, studies conducted to date to determine the effectiveness of physicians' advice have reported mixed results [7-10].

Significant results are difficult to achieve because health-related habits of patients are influenced by deeply rooted socioeconomic, demographic, and cultural factors [1,11]. Perceived health, additional risk factors within the sedentary population, and willingness to change behaviour of inactive primary care populations are key for tailoring interventions and for prioritising physician's counselling efforts [9,12]. Further research is needed to understand the characteristics associated to willingness to change of inactive patients, as some studies have shown the mediating effect of constructs related to the stages of motivational readiness for PA in interventions conducted in primary care [13,14]. In spite of this, a limited amount of research has been carried out on the distribution of these variables within the population to which interventions of family physicians for promoting physical activity are targeted [15,16].

The purpose of this study is to contribute to the development and planning of innovative and feasible interventions to effectively promote PA by describing the clinical and socio-demographic characteristics, willingness to change, and factors associated with readiness to increase PA level in patients categorised as physically inactive by their GPs during a routine visit to their offices.

## Methods

This cross-sectional analysis describes the baseline characteristics of participants in a multi-centre randomised clinical trial conducted in Spain to evaluate the effectiveness of the Experimental Program for Physical Activity Promotion [17]. The trial was approved by the clinical research ethics committee of Galdakao Hospital, Basque Health Service in 2001 (Approval Number: 230901/PI020015).

### Participants

All inactive patients aged 20–80 years attending 56 collaborating general practices at 11 public primary health care centres (see Additional file 1) between October 2003 and May 2004 were eligible to participate in this study. During this period, each GP had to perform the recruitment process one or two days per week. Each physician was expected to recruit 100 participants. Each day research nurses selected 10 patients for each of the physicians who had to perform the recruitment process that day by systematic sampling from the complete list of appointed patients. Research nurses first determined the sampling interval, calculated as the ratio between the total number of patients appointed for that day (sampling frame) and the number to be selected (n = 10) for each GP. Nurses then determined the random starting point using a random digit procedure, and proceeded to systematic selection. Finally, GPs invited to participate in the study all selected patients who attended their offices.

### PA assessment by GPs

After addressing the reason for consultation, physicians interviewed the selected patients to identify those who did not meet PA recommendations [2], guided by an interactive web-based algorithm with the following questions: (1) Do you exercise? (2) What type of exercise and how hard do you exercise (examples of intensity)? (3) How often and for how long do you exercise? Following, additional screen-shots included in the web-based software helped GPs to review the exclusion criteria for the clinical trial with those patients identified as physically inactive: cardiovascular disease, musculoskeletal problems that are exacerbated by exercise, major chronic respiratory, renal, or hepatic disease, an infectious or metabolically unstable condition, severe emotional distress, complicated pregnancy, and follow-up difficulties.

### Measurements

Subjects identified as physically inactive by their family doctor and who consented to participate were referred for measurements, performed by trained research nurses at exercise laboratories installed at each collaborating primary care centre. Data quality was ensured by initial intensive one-week training of research nurses, a pilot study followed by a three-day review training, and double data entry into a centralised Oracle™ database. Quality control was performed daily by online supervision of the study process and data, daily feedback to nurses, monthly progress reports, and regular meetings with the collaborating investigators and nurses every four months.

PA was measured using the *7-day Physical Activity Recall *(PAR) semi-structured interview [18]. The PAR counts time spent in leisure and occupational activities of more than 10 accumulated minutes and different intensities for the 7 days prior to the interview. Minutes per week pertaining to moderate and vigorous PA (min/wk of MVPA) are directly calculated, while activity dose in METs.h/wk is estimated by multiplying the hours devoted to activities of moderate, hard, and very hard intensity by the corresponding metabolic equivalents (METs): 4, 6, and 10 METs respectively. For total energy expenditure in kcal/kg/week, sleeping time and light intensity activity, multiplied by 1 and 1.5 METs respectively, are added to the activity dose. The PAR was the reference standard used to independently confirm if patients identified as physically inactive by the physician did or did not meet the minimum public health PA recommendations, that is, at least 30 min of moderate PA 5 days per week, 20 min of vigorous intensity PA 3 days per week, or hybrid combinations of moderate and vigorous intensity PA episodes [2].

Assessment of PA stage of change, representing ordered categories of motivational readiness to change, was based upon Reed et al recommendations [19]. Participants had to select in a self-administered questionnaire including the abovementioned definition of regular PA [2], the statement best describing their current status from among 5 choices: "No, I do not exercise regularly and I do not intend to do it in the next 6 months" (Precontemplation); "I do not exercise regularly, but I intend to do it in the next 6 months" (Contemplation); "I do not exercise regularly, but I intend to do it in the next 30 days" (Preparation); "I have been exercising regularly for less than 6 months" (Action); "I have been exercising regularly for more than 6 months" (Maintenance). For the analysis of readiness to change, people in the last category were considered misclassified and excluded, because they claimed to exercise regularly but had been confirmed to be inactive by both their physicians and the PAR.

Health-related quality of life measures were obtained using the Spanish version of the Medical Outcomes Trust SF-36 questionnaire (version 1) [20,21]. Standardised scores were calculated for the Spanish population (mean for each sex = 50, standard deviation = 10) [21]. Cardiovascular risk factors were reported by family physicians after reviewing patients' records. Smoking was obtained by self-report and alcohol consumption was identified using the Spanish version of the AUDIT, which defines a risky drinker as a person who scores 8 or more points [22]. Social class and educational level were recorded and classified in accordance with the recommendations of the Spanish Society of Epidemiology [23].

### Statistical Analysis

The positive predictive value of PA assessment by GPs was calculated as the proportion of identified inactive patients who did not meet the minimum recommendations according to the PAR. The prevalence of inactive patients identified by the physician was corrected by this predictive value. All analyses accounted for the clustered structure of data, with patients nested within practices. Descriptive statistics and standard errors were computed using SAS^® ^PROC SURVEYFREQ and SURVEYMEANS (SAS Institute Inc., Cary, NC, USA, 2002). To evaluate the association between characteristics of participants and their readiness to change PA level (precontemplation, contemplation, preparation, and action stages), multivariate mixed effects ordinal logistic regression models were adjusted using SAS^® ^PROC GLIMMIX, with practices as intercept random effects. The contribution of variables included in these models was determined using a generalised score test (significance criterion p < 0.05), and non-significant terms were removed following a backward strategy. Prevalence odds ratios (POR) to have a higher stage of readiness to change and 95% confidence intervals (95% CI) were estimated.

## Results

**Figure 1:**
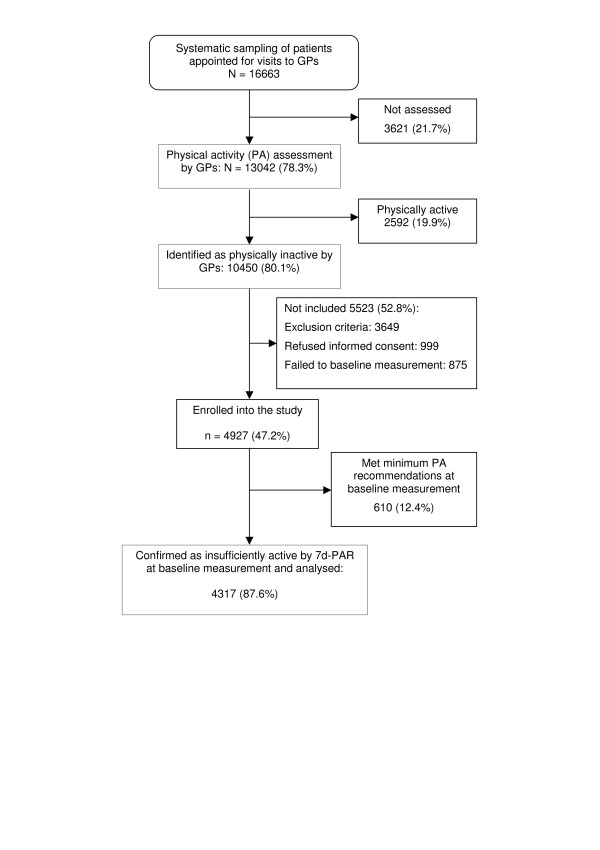
Flowchart of patient recruitment and participation.

**Figure 2:**
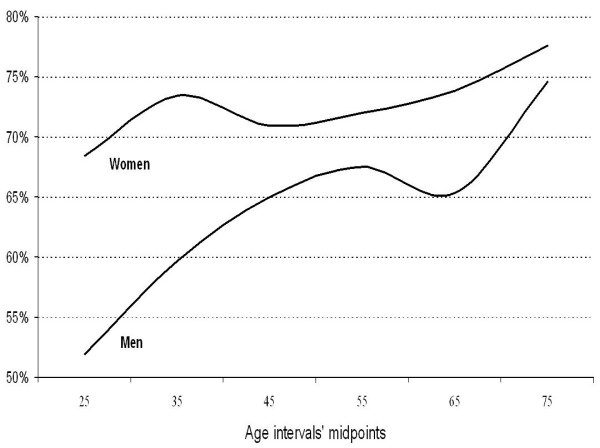
Corrected prevalence of physical inactivity among 13,042 primary care patients in Spain.

Among the 16,663 patients selected, 3,621 were not assessed by their GPs because they did not attend the appointment or due to technical difficulties (i.e. web access problems) or physicians' lack of time. The remaining 13,042 patients (78.3%) were asked the screening questions by their GPs, and 10,450 (80.1%, 95% CI: 77.1% to 83.1%) were identified as inactive and continued the recruitment process. Of these, 3,649 (34.9%) patients met at least one exclusion criterion, 999 (9.5%) refused to participate, 875 (8.4%) failed baseline measurement, and 4,927 were included in study (47.2%). Subsequently, according to the PAR, 12.4% of patients enrolled into the study met the minimum public health recommendations. Thus, the algorithm used by the physicians to identify inactive patients showed an 87.6% positive predictive value, and the corrected prevalence of physical inactivity was at least 70.2% (95% CI: 67.6% to 72.8%) (Figure 1). Physical inactivity increases with age, and its prevalence is higher in women than in men (p < 0.001), especially among younger people (Figure 2).

The following analyses correspond to the 4,317 patients enrolled who did not meet PA recommendations according to both the physician's assessment and PAR. The mean age of inactive patients attending primary care was 50 years (SD = 15), with women (66%) being slightly younger than men (p < 0.01). People working out home (61% of men and 45% of women), from a low social class (53%), and with no university studies (83%) predominated within the sample. Overall, they devoted 33.84 minutes per week to moderate and vigorous physical activity (95% CI, 27.96 to 39.72), which resulted in a weekly activity dose of 2.36 MET.h/week (CI 95%, 1.96 to 2.76). All estimations of PA level were higher for men (p < 0.01). With regard to readiness to change, 62% of patients were in the contemplation and pre-contemplation stages, while 30% considered themselves to be prepared for or attempting a change (Table 1).

**Table 1:** Socio-demographic characteristics, physical activity level, and physical activity stage of change of inactive primary care patients. Values are proportions (95% CI), unless otherwise indicated

	Total N = 4317	Men N = 1484 (34.4%)	Women N = 2833 (65.6%)
Age *mean (95% CI)*	50.0 (49.0 to 51.1)	51.1 (50.0 to 52.2)	49.5 (48.3 to 50.6)
Work Status			
Works out of home	50.6 (46.9 to 54.2)	61.0 (56.7 to 65.4)	45.2 (41.3 to 49.0)
Student	2.1 (1.6 to 2.7)	1.6 (1.00 to 2.3)	2.4 (1.6 to 3.2)
Homemaker	24.0 (21.0 to 27.1)	0.1 (0.0 to 0.3)	36.5 (32.2 to 40.9)
Retired	15.6 (13.3 to 17.8)	29.3 (25.6 to 33.0)	8.3 (5.9 to 10.7)
Unemployed	4.9 (4.2 to 5.6)	4.4 (3.1 to 5.6)	5.2 (4.3 to 6.0)
Other	2.8 (1.9 to 3.6)	3.6 (2.3 to 4.8)	2.4 (1.5 to 3.2)
Educational Level			
None	6.1 (4.7 to 7.5)	4.1 (2.8 to 5.4)	7.2 (5.5 to 8.8)
Elementary School	30.0 (27.1 to 32.9)	28.1 (25.0 to 31.2)	31.0 (27.6 to 34.3)
Middle or High School	47.1 (44.8 to 49.3)	49.4 (46.9 to 51.9)	45.8 (43.0 to 48.6)
University studies	16.8 (14.1 to 19.5)	18.4 (14.8 to 22.0)	16.0 (13.4 to 18.6)
Social Class^a^			
IV–V, Manual worker	52.6 (48.4 to 56.7)	50.4 (46.1 to 54.7)	53.7 (49.3 to 58.1)
III, Intermediate employee	29.7 (27.8 to 31.7)	29.8 (27.1 to 32.5)	29.7 (27.6 to 31.9)
II, Manager small company	10.8 (8.7 to 13.0)	12.3 (9.2 to 15.3)	10.1 (8.0 to 12.1)
I, Manager large company	6.9 (5.1 to 8.7)	7.5 (5.4 to 9.7)	6.5 (4.7 to 8.3)
Physical activity level			
Total Energy Expenditure in Kcal/Kg/day *mean (95% CI)*	32.43 (32.37 to 32.47)	32.48 (32.41 to 32.54)	32.40 (32.34 to 32.45)
Activity dose in MET.h/week *mean (95% CI)*	2.36 (1.96 to 2.76)	2.79 (2.16 to 3.41)	2.14 (1.80 to 2.48)
Minutes/week devoted to moderate and vigorous PA *mean (95% CI)*	33.84 (27.96 to 39.72)	40.53 (31.50 to 49.57)	30.33 (25.36 to 35.30)
PA Stage of Change			
Precontemplation	28.5 (24.0 to 32.9)	28.8 (23.5 to 34.2)	28.3 (24.1 to 32.5)
Contemplation	33.4 (30.5 to 36.3)	32.8 (29.4 to 36.2)	33.7 (30.7 to 36.7)
Preparation	25.7 (22.2 to 29.2)	25.3 (21.2 to 29.3)	25.9 (22.5 to 29.4)
Action	3.9 (3.1 to 4.7)	3.4 (2.3 to 4.4)	4.2 (3.2 to 5.3)
Maintenance	8.5 (6.6 to 10.4)	9.7 (7.3 to 12.1)	7.9 (6.0 to 9.7)

^a ^Social class categorisation based on occupation and work position as recommended by the Spanish Society of Epidemiology (23): Class IV to V includes non-qualified and qualified manual workers; Class III includes the administrative workforce, supervisors and free-lance workers; Class II includes managers of company with less than ten employees, professionals with first level university degree, senior technicians, artists and sportsmen/women; Class I includes managers of public organizations or private companies with more than ten employees, professionals with second and third level university degrees.

The prevalence of at least one risk factor additional to inadequate activity was 85% (95% CI, 83% to 86.3%), and was higher in men than in women (p < 0.001). Only 15% of inactive patients were free of any of these risk factors, while 36% had at least one risk factor in addition to being physically inactive, 30% had two risk factors, and 19% three or more risk factors (28% for men, 95% CI: 24.7% to 31% and 14% for women, 95% CI: 11.8% to 16%). The standardised values of the SF-36 scales suggested that health-related quality of life of inactive primary care patients was below the levels of the Spanish population (<50 points), except for the physical functioning scale (Table 2).

**Table 2:** Risk factors and quality of life of inactive primary care patients. Values are proportions (95% CI), unless otherwise indicated

	Total N = 4317	Men* N = 1484 (34.4%)	Women* N = 2833 (65.6%)
Body mass index (n = 4315)			
Normal (< 25 kg/m^2^)	32.9 (30.5 to 35.3)	23.3 (21.1 to 25.4)	37.9 (34.7 to 41.1)
Overweight	41.2 (39.7 to 42.7)	51.6 (49.5 to 53.7)	35.8 (33.8 to 37.8)
Obese (≥ 30 kg/m^2^)	25.9 (23.9 to 27.9)	25.1 (22.9 to 27.3)	26.3 (23.7 to 28.9)
Smoking			
Current smoker	30.4 (28.5 to 32.2)	37.3 (34.4 to 40.1)	26.8 (24.9 to 28.6)
Former smoker	18.8 (17.2 to 20.5)	31.9 (29.0 to 35.0)	12.0 (10.4 to 13.5)
Never smoker	50.8 (48.5 to 53.0)	30.8 (28.3 to 33.3)	61.2 (58.7 to 63.8)
Hypertension	25.2 (22.8 to 27.6)	28.7 (25.8 to 31.6)	23.4 (20.8 to 26.0)
Dyslipidemia	21.1 (17.5 to 24.6)	24.5 (20.3 to 28.6)	19.3 (15.8 to 22.7)
Diabetes	8.5 (7.5 to 9.6)	12.0 (10.2 to 13.8)	6.7 (5.7 to 7.7)
Risky drinker (n = 4248)	5.1 (4.1 to 6.2)	12.0 (9.5 to 14.4)	1.5 (1.0 to 2.0)
Number of risk factors in addition to physical inactivity (n = 4246)			
0	15.5 (13.7 to 17.2)	8.3 (6.7 to 9.8)	19.2 (17.0 to 21.4)
1	36.2 (34.3 to 38.1)	30.3 (27.6 to 33.0)	39.3 (37.2 to 41.3)
2	29.6 (28.1 to 31.2)	33.6 (31.1 to 36.1)	27.6 (25.5 to 29.6)
3	18.7 (16.6 to 20.9)	27.8 (24.7 to 31.0)	13.9 (11.8 to 16.0)
Health-related quality of life^a ^*mean (95%CI)*			
Physical function (n = 4308)	50.7 (50.2 to 51.2)	50.3 (49.9 to 50.8)	50.8 (50.3 to 51.4)
Role physical (n = 4300)	46.3 (45.4 to 47.2)	45.9 (44.8 to 46.9)	46.5 (45.6 to 47.4)
Bodily pain (n = 4315)	44.4 (43.8 to 45.0)	43.5 (42.8 to 44.2)	44.9 (44.3 to 45.6)
General health (n = 4304)	48.5 (48.0 to 49.0)	47.9 (47.3 to 48.4)	48.8 (48.3 to 49.4)
Vitality (n = 4308)	46.9 (46.3 to 47.5)	47.7 (47.1 to 48.2)	46.5 (45.8 to 47.1)
Social functioning (n = 4313)	47.9 (46.9 to 48.9)	47.8 (46.7 to 48.9)	48.0 (47.0 to 49.1)
Role emotional (n = 4300)	46.4 (45.7 to 47.1)	46.2 (45.4 to 47.0)	46.6 (45.7 to 47.5)
Mental health (n = 4306)	46.8 (46.2 to 47.3)	47.4 (46.8 to 48.0)	46.4 (45.8 to 47.0)

* All sex comparisons were significant at p < 0.01 except for Role Physical, Social Functioning and Role Emotional scales of the SF-36.

^a ^Standardised by sex norm-based scores relative to the Spanish population (mean of 50 and standard deviations of 10 points) (21).

According to the multivariate ordinal logistic regression analyses summarised in Table 3, after simultaneously controlling for the effect of the remaining variables included in the model, a younger age, not working out of home, a higher educational and social class level, obesity, and hypertension increased the odds of having a higher stage of motivational readiness to change (p < 0.05). Score test was consistent with the proportional odds assumption of the ordinal model (p = 0.18).

**Table 3:** Characteristics associated with motivational readiness to change physical activity level (n = 3940)

Final multivariate adjusted ordinal logistic model for stage of change		
Probability of readiness to change at reference levels of independent covariates (95%CI):		
Contemplation to action stage	31.8% (22.8% to 42.4%)	
Preparation or action stage:	7.5% (4.9% to 11.4%)	
Action stage:	0.7% (0.4% to 1.1%)	

Independent covariates	Adjusted POR (95% CI)	p-value

Age^a^		<0.001
70 to 80	1.00	
60 to 69	1.97 (1.53 to 2.53)^a^	
50 to 59	2.65 (1.86 to 3.77)^a^	
40 to 49	3.17 (2.12 to 4.73)^a^	
30 to 39	3.35 (2.22 to 5.05)^a^	
20 to 29	3.14 (2.10 to 4.69)^a^	
Work Status		0.033
Works out of home	1.00	
Student	1.36 (0.89 to 2.09)	
Homemaker	1.31 (1.10 to 1.55)	
Retired	1.26 (1.00 to 1.60)	
Unemployed	1.20 (0.92 to 1.59)	
Other	1.15 (0.80 to 1.65)	
Education Level		0.003
None	1.00	
Elementary School	1.34 (1.02 to 1.77)	
Middle or High School	1.61 (1.20 to 2.17)	
University studies	1.87 (1.32 to 2.65)	
Social Class		<0.001
IV–V, Manual worker	1.00	
III, Intermediate employee	1.32 (1.15 to 1.52)	
II, Manager small company	1.17 (0.93 to 1.46)	
I, Manager large company	1.52 (1.15 to 2.00)	
Risk Factors		
Obesity	1.22 (1.03 to 1.45)	0.02
Hypertension	1.23 (1.01 to 1.50)	0.04
Obesity plus Hypertension	1.10 (0.90 to 1.35)	*

^a ^Effect of Age + Age^2 ^estimated at age intervals' midpoints.

* P-value for the interaction between Obesity and Hypertension = 0.038

## Discussion

This study shows that when GPs assess PA status and readiness to change, they find that the great majority of primary care patients (more than 70%) do not meet the minimum recommended levels, and most of them report they are not immediately ready to change (more than 62% are in the precontemplation and contemplation stages). These results together with inconclusive evidence for physician's advice [7,10] support the need for a targeted strategy for PA promotion in primary care settings. It is clear that under the circumstances currently prevailing in most healthcare systems, it is not possible for primary care physicians to implement PA interventions to all their inactive patients [24].

Detection of inactive patients is simplified by the use of the three brief questions asked in this study, which have a high positive predictive value (88%). However, merely asking about PA will not lead to a change in behaviour. Minimal interventions can be reduced to less than 3 minutes, but most primary care physicians do not have enough time to implement the intervention with all their sedentary patients [24]. A reasonable approach would be to select as target population those patients who might benefit more, e.g. those who are more ready to change, those with current health problems, and those who are at a high risk for developing health problems [9].

Adaptation of the behavioural counselling interventions conducted by the family physician to the motivation, characteristics, and needs of the patient is recommended [13,14]. Certain socio-demographic characteristics associated in this study with motivation for change might be useful for GPs to prioritise time, effort, and targeting intervention strategies to specific population subgroups who would like to change their sedentary behaviour. Patients aged less than 50 years old had a three times greater probability of having a higher motivation to increase PA as compared to those over 70 years of age. After controlling for the effect of age and all other covariates, being retired and working at home were positively associated with motivational readiness to change PA as compared to people who worked out of home. This may partly be due to the fact that retired people and homemakers have more time available as compared to people with competing work obligations. The higher the educational level and social class, the more likely a higher motivational readiness to change PA. The combination of these socio-demographic characteristics markedly increases the probability that patients are ready to increase their level of physical activity. Advising and assisting the subgroup of more motivated sedentary patients would not require busy practitioners to invest the same time as for a strategy directed to all inactive patients. However, this strategy would discriminate against more disadvantaged patients. The most frequent socioeconomic characteristics of inactive patients attending family practices, such as having a low educational level or pertaining to the manual working class, are associated with a lower probability of being ready to change. Previous studies have reported a lack of motivation to modify the level of physical activity associated with advanced age and a low educational level [25-27]. Further research of these associations is required to find out optimal interventions for motivating people not ready to change their PA level.

An additional criterion for selecting the target population for physician interventions would take into account the relevance of PA for a number of risk factors and for health-related quality of life. While the proportion of smokers in our sample was similar to the general population (31.8% of smokers in the Spanish population), the prevalence of obesity, hypertension, dyslipidemia, diabetes, and risky drinking was approximately two times greater (13.3%, 12.2%, 8.9%, 5%, and 2.4% respectively, in the community) [28]. Most patients in the sample had at least one risk factor in addition to inadequate activity level and their quality of life was below the standard in the community [21]. This is consistent with literature about clustering of risk factors [15,16,29] and describes a population in which PA promotion is truly justified considering its potential benefits [1,2,30,31]. The association of low activity levels, poor quality of life, and risk of morbidity and mortality may be a strong argument to support interventions for PA promotion [9]. In this study, the probability of being ready to change PA level was higher in obese patients and those diagnosed of hypertension. However, combination of both risk factors in addition to inactivity cancels this effect.

### Strengths and limitations

For an adequate interpretation of our results, some methodological comments are needed. The analyses reported here describe a selected population characterised by very low levels of physical activity. Patients were slightly more inactive than those in clinical trials conducted with primary care sedentary populations [15,16,31,32], and clearly less physically active than the overall population of the community (58.4% of which is estimated to be physically active) [28]. Though people with cardiovascular diseases were excluded from the study, the systematic sampling used for selecting eligible patients, together with their detection conducted by 56 GPs under routine practice conditions in 11 cities from 8 different autonomous regions in Spain, give the results a great capacity for generalisation to insufficiently active patients seen in primary care. Nevertheless, the cross-sectional nature of these analyses does not allow for interpreting the directionality of the relationship between physical inactivity, clustering of risk factors, and lower quality of life.

The use of self-reporting for PA measurement represents another limitation of the study, as it has been suggested that sedentary individuals tend to overestimate the intensity of their activities, especially recalling moderate intensity activities, when using self-report instruments [33]. However, recent validity studies have concluded that the 7-day PAR provides a reasonable estimate of physical activity energy expenditure [34]. Patients' self-categorisation in a physical activity stage of change also depends on self-perception, and may therefore reflect perceived rather than actual motivational readiness [35].

## Conclusion

Our results have significant implications for primary care services. The high proportion of inactive patients, the poor motivation of most of them, and the lack of strong evidence for physician counselling support a selective rather than an overall population approach to PA promotion in primary care. The questions used in this study to detect insufficiently active people in primary care are appropriate because of their high predictive value. After asking and briefly advice all patients about PA, selection of a target population based on inactive patients' readiness to change is recommended. For this, GPs may offer an additional appointment to prescribe a PA plan, which will result in self-selection of those attending that extra appointment. This would notably reduce the target population to a more acceptable size for time-pressured GPs, selecting for PA prescription those who are more willing to change. Evidence for the effectiveness of this strategy will result from the clinical trial conducted to evaluate the Experimental Programme for Physical Activity Promotion [17]. In addition to this, a second selective criterion would consider the clustering of risk factors and chronic conditions.

This selective approach may help GPs prioritise PA promotion efforts from a logical, practical, and sustainable perspective. However, it may discriminate against older people and those with low educational and social class levels, as these are characteristics associated with a lower motivation. In addition, limitations of individual interventions are increasingly apparent, and a more comprehensive social-ecological model that goes beyond the exclusive domain of patient-practitioner interaction is required, particularly for less motivated people [36,37]. In this approach, the health system plays a major role, but complementary to other individual, cultural, and social factors conditioning healthy lifestyles. Nevertheless, evidence to support all those statements still needs to be obtained [9].

## Competing interests

The authors declare that they have no competing interests.

## Authors' contributions

GG conceived the idea for the study. He led on study design, planning, project coordination, analysis, interpretation of results, and writing of the article. AS coordinated the field work, was responsible for quality control of the study process, data management, and collaborated in the analysis and interpretation of results, writing, and critical review of the article. ROS–P and JT were responsible for training of physicians and standardisation of the clinical protocol. They participated in interpretation of results, writing and critical review of the article. KL and JS were responsible for training research nurses, provided important advice regarding the measurement of physical activity and critically reviewed the article. GG, AS, ROS–P, JT, KL, and JS cooperated in the study design and obtained funding. 

All members of the PEPAF group listed in the Appendix coordinated the study in each collaborating centre, collected data, and critically reviewed the article. All contributors approved this version submitted for publication to the journal BMC Public Health. Name of guarantor: Gonzalo Grandes

## Pre-publication history

The pre-publication history for this paper can be accessed here:



## Supplementary Material

Additional file 1 Appendix: PEPAF group research collaborators. Click here for file
