# Supplementary material for: Targeting physical activity promotion in general practice: Characteristics of inactive patients and willingness to change
Source: BMC Public Health. 2008 May 22;8:172. doi: 10.1186/1471-2458-8-172 (PMC2412873; doi:10.1186/1471-2458-8-172)
Supplement: Additional file 1 — Appendix: PEPAF group research collaborators. [file 1471-2458-8-172-S1.doc]

**Appendix: PEPAF group research collaborators.**

**Director Team – Primary Care Research Unit of Bizkaia, Basque Health Service-Osakidetza.** Principal Investigator: Gonzalo Grandes; Research Team: Jesús Torcal, Ricardo Ortega, Alvaro Sánchez, Kepa Lizarraga, Javier Serra, Imanol Montoya. **From Basauri-Aríz Health Centre, Basque Health Service-Osakidetza.** Family Physicians: Ángel Fernández, Victor Manuel López, Lourdes Marijuán, Begoña Etxeguren, Victor Landa, Jesús Martínez, Mª Mercedes Díez, Juan Ramón Lejarza; Research Nurse: Judith González. **From Galdakao Health Centre, Basque Health Service-Osakidetza.** Family Physicians: Vidal Salcedo, Idoia Ibáñez, Agurtzane Ortego, Pedro Iraguen, Pilar Echevarria; Research Nurse: Virginia Villaverde. **From Algorta Health Centre, Basque Health Service-Osakidetza.** Family Physicians: Amaia Ecenarro, Mª Teresa Uribe, Carmen Moral, Eguskiñe Iturregui; Research Nurse: Ana Belén Alonso. **From Camas Health Centre, Andalucia Health Service (Sevilla).** Research Team Head: José María Páez; Family Physicians: Mª Ángeles Tarilonte, Concepción Molina, Vicente Rodríguez, Isabel Villafuente. Research Nurse: Mercedes Álvarez. **From Dalt Sant Joan Health Centre Balear Island Health Service (Mahón).** Research Team Head: Andreu Estela; Family Physicians: Txema Coll, Angels Llach, Josep Mª Masuet, Ana Moll; Research Nurse: Monica Pons. **From Serraparera Health Centre, Catalonian Health Service (Barcelona).** Research Team Head: Bonaventura Bolibar; Family Physicians: Agusti Guiu, Amadeu Díaz, Xavier Martínez, Mª Dolores Hernández, José Ignacio Olivares, Francisco Hernansanz, Rita Ayala; Research Nurse: Ana Cascos. **From San Fernando Cuenca III Health Centre, Castilla y la Mancha Health Service (Cuenca).** Research Team Head: Vicente Martínez; Family Physicians: Mª del Carmen García, Mª Ángeles Gabriel, Mª Luscinda Velázquez, Natividad Ortega, Mª Jesús Segura; Research Nurses: Rodrigo Cerrillo, Patricia López. **From La Alamedilla Health Centre, Castilla y León Health Service (Salamanca).** Research Team Head: Luis García; Family Physicians: José Antonio Iglesias, Manuel Gómez, Emilio Ramos, Pilar Moreno; Research Nurses: Yolanda Castaño, Nadia Carrillo. **From Casa Barco Health Centre, Castilla y León Health Service (Valladolid).** Research Team Head: Carmen Fernández; Family Physicians: Amparo Gómez, Miguel Angel Díez, Ruperto Sánz, Luis Miguel Quintero; Research Nurse: Jose Ignacio Recio. **From Sardoma Health Centre, Galicia Health Service (Vigo).** Research Team Head: Pilar Gayoso; Family Physicians: Luciano Casariego, Manuel Domínguez, Jose Ramón Moliner, Fernando Lago, Mª Concepción Cruces; Research Nurse: Marisa Enríquez. **From Guayaba Health Centre, Madrid Health Service (Madrid).** Research Team Head: Tomás Gómez Gascón; Family Physicians: Javier Martínez, José Antonio Granados, Mª Ángeles Fernández, Mª Isabel Gutiérrez, Carlos San Andrés, Concepción Vargas-Machuca; Research Nurse: Cristina Díaz.
